# The Evaluation of Nosocomial Infections in Pediatric Patients with Extracorporeal Membrane Oxygenation Support

**DOI:** 10.21470/1678-9741-2017-0072

**Published:** 2017

**Authors:** Pelin Ayyıldız, Taner Kasar, Erkut Ozturk, Okan Yildiz, Serpil Ozturk, Yakup Ergul, Sertac Haydin, Alper Guzeltas

**Affiliations:** 1 Department of Pediatric Cardiology, Istanbul Saglik Bilimleri University Istanbul Mehmet Akif Ersoy Thoracic and Cardiovascular Surgery Education and Research Hospital, Istanbul, Turkey.; 2 Department of Cardiovascular Surgery, Istanbul Saglik Bilimleri University Istanbul Mehmet Akif Ersoy Thoracic and Cardiovascular Surgery Education and Research Hospital, Istanbul, Turkey.; 3 Department of Infectious Diseases, Istanbul Saglik Bilimleri University Istanbul Mehmet Akif Ersoy Thoracic and Cardiovascular Surgery Education and Research Hospital, Istanbul, Turkey.

**Keywords:** Extracorporeal Membrane Oxygenation, Cross Infection, Child

## Abstract

**Introduction:**

Extracorporeal membrane oxygenation (ECMO) has become a standard technique over the past few decades in intensive care unit (ICU).

**Objective:**

A review of pediatric patients who received ECMO support in the pediatric cardiac ICU was conducted to determine the incidence, risk factors and causal organisms related to acquired infections and assess the survival rates of ECMO patients with nosocomial infections.

**Methods:**

Sixty-six patients who received ECMO support in the pediatric cardiac ICU between January 2011 and June 2014 were included in the study. Demographic, echocardiographic, hemodynamic features and surgical procedures were reviewed.

**Results:**

Sixty-six patients received a total of 292.5 days of venoarterial ECMO support. Sixty were postoperative patients. Forty-five patients were weaned from ECMO support with an ECMO survival rate of 68.2%. The rate of infection was 116.2/1000 ECMO days. Prolonged ICU stay, duration of ventilation and ECMO were found associated with development of nosocomial infection and only the duration of ECMO was an independent risk factor for nosocomial infections in ECMO patients.

**Conclusion:**

The correction of the underlying process leading to ECMO support and shortening the length of ECMO duration together with stricter application of ECMO indications would improve the infection incidence and hospital surveillance of the patient group.

**Table:** 

Abbreviations, acronyms & symbols	
ACT	= Activated clotting time
aPTT	= Activated partial thromboplastin time
BSI	= Blood stream infections
BUN	= Blood urea nitrogen
CDC	= Centers for Disease Control
CRP	= C-reactive protein
ECMO	= Extracorporeal membrane oxygenation
ELSO	= Extracorporeal Life Support Organization
ICU	= Intensive care unit
RACHS-1	= Risk-Adjusted Congenital Heart Surgery
RTI	= Respiratory tract infection
SWI	= Sternal wound infections
UTI	= Urinary tract infection
VA	= Venoarterial

## INTRODUCTION

The use of extracorporeal membrane oxygenation (ECMO) has become a standard technique to provide temporary respiratory and cardiovascular support to pediatric and adult patients over the past few decades^[1]^. Despite the efficacy, significant associated risks including infections are present^[2]^. The cannulation of major vessels provides entry for infectious agents along with additional invasive devices such as urinary catheters, endotracheal tubes, dialysis and central venous catheters, which further increase the risk of nosocomial infection in patients on ECMO^[3,4]^.

ECMO support has been used in our pediatric cardiology and cardiovascular intensive care unit (ICU) and the patient records who have received ECMO were retrospectively collected. This study aims to determine the incidence, risk factors and causal organisms related to acquired infections in pediatric patients who received ECMO and assess the survival rates of ECMO patients with nosocomial infections.

## METHODS

The study was a retrospective cohort study and approved by the Institutional Review Board. Written consent was taken from the parents.

Sixty-six patients who received ECMO support in the pediatric cardiac ICU at the Mehmet Akif Ersoy Thoracic and Cardiovascular Surgery Center between January 2011 and June 2014 were included in this retrospective study. The medical records were retrospectively reviewed from database of the hospital and incomplete data was fulfilled from individual chart review; age, sex, underlying medical condition, length of stay in the pediatric ICU, length of hospitalization, duration of ventilation, indication for ECMO support, duration of ECMO support, duration of post ECMO ventilation, empiric antibiotics within first 24 hours of ECMO, ECMO-related infections, type of infection and causative agents, pH, C-reactive protein (CRP), blood urea nitrogen (BUN), lactate before ECMO support, and final outcome were recorded. During the study period, there was no defined protocol for antibiotic prophylaxis for ECMO in our clinic.

A hospital-acquired infection was defined as an infection that was not present, nor an extension of an infection present, on admission to the hospital. All hospital-acquired infections including blood stream infections (BSI), respiratory tract infection (RTI), urinary tract infection (UTI) and sternal wound infections (SWI) of the patients were recorded and Centers for Disease Control and Prevention (CDC) criteria were used as standard definitions for ECMO related hospital-acquired infections^[5]^. Nosocomial infections that occurred 24 h after initiation and 48 h after discontinuation of ECMO were defined as ECMO-related nosocomial infections^[6]^. The percentage of patients who survived after ECMO discontinuation to the total number of patients who received ECMO was defined as the ECMO survival rate, and the percentage of patients who survived to discharge from hospital to the total number of patients who received ECMO was defined as the overall survival rate^[6]^.

All patients were intubated before ECMO. Since our patient population was mainly postoperative cardiac patients, they all underwent venoarterial (VA) cannulation. DLP (Medtronic^®^, Inc., Minneapolis, MN, USA) arterial and venous ECMO cannula were used for ECMO cannulation for all patients.

A central venous line and a nasogastric tube were placed before ECMO was initiated. Medos Deltastream^®^ System has been in use for ECMO support in our ICU unit and after November 2012, Medos Deltastream^®^ DC system with DP2 pump head (MedosAG, Stolberg, Germany) was switched to the Medos Deltastream^®^ MDC system with DP3 pump head (MedosAG, Stolberg, Germany). Continuous intravenous heparin infusion was started and titrated at a dosage adequate to keep activated clotting time (ACT) around 180-200 sec and activated partial thromboplastin time (aPTT) in a range of 60-80 sec. The ECMO pump flow started at a rate of 100 ml/kg/min but higher flow of 150-200 ml/kg/min was preferred in patients with single ventricle and after shunt operations with sternotomy. The flow rate was set again after the correction of end-organ perfusion, lactic acidosis, arterial blood gases and an increase in systemic venous oxygen saturation was established. Urine cultures were obtained weekly and when clinically indicated. Patients were removed from ECMO when their cardiac or pulmonary status improved or they were decannulated because of irreversible disease as severe neurologic injury^[4]^.

### Statistical Analysis

All analyses were performed using SPSS 15.0 for Windows (SPSS, Chicago, IL, USA). The statistical significance of continuous variables was determined using nonparametric tests (the Mann-Whitney U-test), and categorical variables were analyzed with Fisher's exact test. A value of *P*<0.05 was considered statistically significant. The median (maximum-minimum) of the variables was reported. After univariate analysis with selected variables, a logistic regression model was used for multivariate analysis to determine the independent predictive factors of ECMO-related infections.

## RESULTS

A total of 66 patients received a total of 292.5 days of VA-ECMO support in the pediatric cardiac ICU. Sixty were postoperative patients, and six patients were internalized by different indications. The Risk-Adjusted Congenital Heart Surgery (RACHS-1) category of the operated patients were median 3 (range 2-6) whereas the O`Brian classification of the operated patients were median 3 (range 1-5). Twenty-five patients were newborns (37.8%), 37 were infants (56%), three were children older than two years of age (4.5%) and one was an adolescent (1.5%). The indications for ECMO were cardiac arrest in 21 patients, hypotension resistant to medication in 36 patients, failure to be separated from cardiopulmonary bypass in four patients, pulmonary hypertensive crisis in two patients, and other in three patients.

Forty-five patients were weaned from ECMO support with an ECMO survival rate of 68.2%. The overall survival rate was 33.3%. All but two deaths occurred more than 48 h after separation from ECMO (Table 1).

**Table 1:** Comparison of patient characteristics with and without infectious complications.

	Total (n=66)	Non-infected (n=38)	Infected (n=28)	*P*
Age (month)	4 (0.03-240)	5.2 (0.03-108)	1.5 (0.03-240)	0.256
Sex (male/female)	38/28	24/14	14/12	0.799
Postoperative ECMO (n)	60	35	25	0.693
Duration of ECMO (day)	4 (1-13)	3.5 (1-8)	4 (1-13)	0.030
pH	7.18 (6.9-7.5)	7.14 (6.9-7.5)	7.19 (6.9-7.43)	0.410
Lactate (mmol/L)	9.8(1.6-129)	10.4(1.6-129)	9 (3.3-17.6)	0.284
CRP (mg/dl)	17 (0.5-185)	19.4 (0.6-116)	17 (0.5-185)	0.870
BUN (mg/dl)	15(2-40)	15(2-38)	14.5(2-40)	0.732
Antibiotics on ECMO day 1	65 (98%)	37 (97,4%)	28 (100%)	0.287
Duration of ICU stay (day)	14.5 (2-114)	11.5 (2-114)	17 (7-81)	0.045
Duration of ventilation (day)	12 (1-114)	9 (1-114)	14.5 (3-66)	0.040
Outcome				
ECMO survival	45 (68.2%)	24 (63.2%)	21 (75%)	0.424
Overall survival	22 (33.3%)	11 (28.9%)	11 (39.3%)	0.435

BUN=blood urea nitrogen; CRP=C-reactive protein; ICU=intensive care unit

A total of 28 patients out of the 66 patients experienced 34 infectious episodes during ECMO support. Culture-positive infections were detected at a single site in 22 patients, and multiple sites in six patients. There were 13 (37.2%) BSI, 10 (29.4%) RTI, 9 (25.7%) UTI, and 2 SWI (5.7%) (Table 2). The nosocomial infection rate was 116.2/1000 ECMO days.

**Table 2:** The characteristics of patients who had culture positive nosocomial infection.

Patient nº	Age	Number of sites of infection	Site of positivity	Microorganism	Days of ECMO	Antibiotics at ECMO initiation	ECMO survival	Result
1	4 months	1	BSI	MRCNS	4	Cefazoline	Exitus	Exitus
2	10 months	1	UTI	*Candida*	4	Cefazoline	Survived	Exitus
3	1 day	1	BSI	MSSA	8	Ampicillin + Cefotaxime	Exitus	Exitus
4	10 days	2	BSI, RTI	*Klebsiella*	3	Ampicillin + Cefotaxime	Survived	Exitus
5	18 days	2	BSI, RTI	*Acinetobacteria*	8	Other	Exitus	Exitus
6	4 months	1	UTI	*Candida*	7	Other	Survived	Exitus
7	24 days	1	RTI	*Pseudomonas*	2	Cefazoline	Survived	Exitus
8	2 days	1	BSI	MRCNS	2	Ampicillin + Cefotaxime	Survived	Exitus
9	21 day	2	BSI UTI	*Micrococcus Candida*	13	Other	Survived	Discharged
10	20 years	1	BSI	MSCNS	2	Other	Survived	Discharged
11	28 months	1	BSI	*Corynebacterium bovis*	2	Cefazoline	Survived	Discharged
12	16 days	1	BSI	*Enterobacteria cloacae*	3	Cefazoline	Exitus	Exitus
13	3 days	2	BSI Tissue	MRCNS *Candida Alb*	3,5	Other	Survived	Exitus
14	13 months	1	BSI	MSCNS	5	Cefazoline	Survived	Exitus
15	1 month	1	BSI	*Stenotrophomonas*	11	Cefazoline	Exitus	Exitus
16	14 days	1	RTI	*Pseudomonas*	2	Cefazoline	Survived	Discharged
17	2 months	1	UTI	*Candida*	7	Cefazoline	Survived	Exitus
18	11 months	1	RTI	*Klebsiella*	4	Cefazoline	Survived	Discharged
19	11 days	2	BSI UTI	*Klebsiella Candida*	7	Ampicillin + Cefotaxime	Exitus	Exitus
20	28 days	1	RTI	*Acinetobacteria*	10	Ampicillin + cefotaxime	Survived	Exitus
21	6 months	1	RTI	*Pseudomonas*	1	Ampicillin + Cefotaxime	Survived	Discharged
22	3 months	2	UTI Tissue	*Candida* MRCNS	4	Ampicillin + Cefotaxime	Survived	Discharged
23	14 months	1	RTI	*E Coli*	4	Cefazoline	Survived	Discharged
24	10 months	1	UTI	*Candida*	8	Cefazoline	Survived	Exitus
25	4.5 months	1	UTI	*Candida*	13	Ampicillin + Cefotaxime	Exitus	Exitus
26	21 days	1	UTI	*Candida*	8	Cefazoline	Survived	Discharged
27	7 months	1	RTI	*Stenotrophomonas*	6	Cefazoline	Survived	Discharged
28	8 days	1	RTI	*Klebsiella*	3	ampicillin + Cefotaxime	Survived	Discharged

BSI=blood stream infection; RTI=respiratory tract infection; UTI=urinary tract infection; MRCNS=meticillin-resistant coagulase negative *staphylococcus*; MSCNS=meticillin-sensitive coagulase negative *staphylococcus*; MSSA=meticillin-sensitive *staphylococcus aureus*

Empiric cefazolin treatment was started in 41 patients and ampicillin-cefotaxime therapy in 14 patients. Ten patients received different combinations of antibiotics due to infections reported before ECMO support (four patients received vancomycin and meropenem, two patients received vancomycin and ceftriaxone, two patients received ceftriaxone, and two patients received sulbactam-ampicillin). One patient received no antibiotics before ECMO support.

Gram-negative bacteria accounted for 44.1% (15/34), Gram-positive bacteria for 26.5% (9/34), and *Candida* 29.4% (10/34) of the total culture-positive pathogens. *Candida* was the most common isolate (29.4%; 10/34) in the ECMO patient population followed by species of *Coagulase-negative Staphylococci* (17.6%; 6/34), *Klebsiella* (14.7%; 5/34), *Pseudomonas* (8.8%; 3/34), *Acinetobacter* (8.8%; 3/34), *Stenotrophomonas maltophilia* (5.9%; 2/34), *Staphylococcus aureus* (2.9%; 1/34), *Micrococcus* (2.9%; 1/34), *Corynebacterium bovis* (2.9%; 1/34), *Enterobacter cloaca* (2.9%; 1/34), and *Escherichia coli* (2.9% (1/34). The subspecies were *Candida Albicans* in eight patients and *Candida Parapsilosis* in the remaining two patients.

**Fig. 1:**
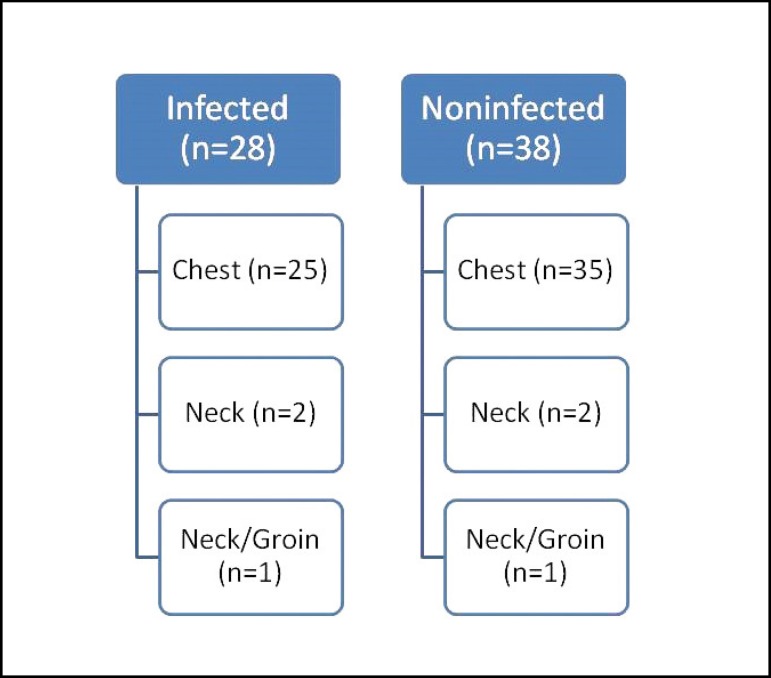
The characteristics of cannulation sites of infected and non-infected patients.

ECMO cannulations were performed through the chest in 60 patients, from the neck in four patients, and from the neck and groin in two patients. No statistically significant difference was found between the cannulation sites as means of infection (*P*=0.925) (Figure 1).

The chest was left open in 61 patients, the skin was closed with silastic patch or primarily in 37 and 24 patients, respectively. Culture-positive infections were observed in 14 of the 24 skin primarily-closed patients (58.3%), but only in 11 of 37 skin patchclosed patients (29.7%). The results of ECMO patients who had skin patch-closed and primarily closed were demonstrated in Table 3. The age and infection rates of the skin patch-closed patients were found significantly lower although the risk categories were significantly higher; the ECMO and mechanical ventilation durations and ICU stay were significantly prolonged in skin primarily-closed patients (*P*< 0.05).

**Table 3:** Comparison of patient characteristics with skin patch-closed and primarily closed.

	Skin-patch closed (n=37)	Skin-primarily closed (n=24)	*P*
Overall survival	10 (27%)	11 (46%)	0.171
ECMO survival	23 (62%)	20 (83%)	0.090
Risk classification (O'Brian)	4 (1-5)	3 (1-5)	0.040
Infected/noninfected	26/11	14/10	0.025
AFR positive/negative	23/14	14/10	0.790
Multiple revisions	22 (59%)	16 (67%)	0.601
Peritoneal dialysis	8 (21%)	6 (25%)	0.765
Hemodialysis	13 (35%)	10 (42%)	0.787
Age (month)	1.5 (0.1-17)	7 (0.1-108)	0.030
Sex (male/female)	20/17	15/9	0.600
Postoperative ECMO (n)	36 (97%)	22 (92%)	0.556
Duration of ECMO (day)	3 (1-8)	4.5 (1-13)	0.040
pH	7.18 (6.9-7.4)	7.19 (6.7-7.5)	0.532
Lactate (mmol/L)	10 (1.6-24)	9.3 (2.2-29)	0.727
BUN (mg/dl)	16.5 (2-40)	14 (2-38)	0.372
Duration of ICU stay (day)	11 (2-114)	21 (4-90)	0.050
Duration of ventilation (day)	9 (1-114)	18.5 (4-66)	0.040

Forty patients were re-operated for bleeding at least once and had been supported by excessive blood products during ECMO where 19 (47.5%) of them had culture-positive infections. In terms of infectious outcomes, no statistically significant difference of culture positivity rates was found between the patients with high rate of bleeding and others (*P*=0.322).

In comparison with Medos Deltastream^®^ DP2 and DP3 systems (25 patient *vs.* 41 patients) in terms of infectious outcomes, no statistically significant difference of culture positivity rates was found (32%, DP2 *vs.* 41%, DP3, *P*>0.05).

When the infected and non-infected patient groups were compared in univariate analysis, prolonged ICU stay, duration of ventilation, and duration of ECMO were associated with the development of nosocomial infection in patients who received ECMO support (*P*=0.045, *P*=0.030, *P*=0.040, respectively). However, multivariate logistic regression analysis revealed that only the duration of ECMO was an independent risk factor for nosocomial infections in patients who received ECMO support (odds ratio:1.318; 95% CI: 1.066-1.63; *P*=0.011). The ratio of patients who were successfully weaned and discharged were not different between infected and uninfected patients (*P*=0.424, *P*=0.435; respectively).

## DISCUSSION

In this study, the characteristics and frequency of nosocomial infections associated with ECMO support in pediatric cardiac patients were described, who are mostly postoperative in a government hospital with a high surgical volume in a developing country.

ECMO has been increasingly used in postoperative pediatric cardiac patients who have inability to wean from cardiopulmonary bypass and have certain risk factors such as infection/sepsis, bleeding, neurologic sequela, etc. The reported rate of infection in these patients varies between 3.5% and 45% in the literature, which might be due to different study designs^[3-8]^.

In 2010, Extracorporeal Life Support Organization (ELSO)^[9]^ reported that 18.7% of all pediatric respiratory ECMO cases were complicated by culture-proven infections whereas in 2011 ELSO Task Force on Infections and ECMO^[2]^ reported a prevalence of 13.7% and a rate of 20.7/1000 ECMO days for culture-proven infections acquired during ECMO in the pediatric cardiac patient population.

Around 42% (28/66) of our patients experienced at least one nosocomial infection during ECMO support with a nosocomial infection rate of 116.2/1000 ECMO days. As emphasized in many studies^[3,4,10]^, the ECMO population in pre/postoperative cardiac failure or arrest, such as the patient population in our study, are among the most critically ill patients.

These patients were also exposed to invasive procedures before and during ECMO, and thus, broad-spectrum antibiotics were frequently prescribed. In addition, the patients required prolonged use of endotracheal intubation tubes, central venous lines, urinary catheters along with the deleterious effects of cardiac surgery. Moreover, entry into the ECMO circuit for laboratory evaluations or for intravascular infusions increases the entry of colonized organisms in the bloodstream, which contributes to the high rate of infections in these patients. The reported decrease in the number and function of white blood cells in ECMO patients with an altered immune response after cardiopulmonary bypass might also add to the increased susceptibility to infections^[11-14]^.

Prolonged ECMO support, ECMO support for cardiac disease in particular apart from other reasons for ECMO, and requiring ECMO support with an open chest were reported as risk factors for the development of infection and especially closed *versus* open chest was reported to be protective in cardiac patients^[3,4,7]^.

Since nearly all of our patients required ECMO with an open chest, we started to close the skin primarily of these patients during ECMO support in our clinic. However, when we evaluated the culture-positive infection difference between the skin-primarily closed and patch-closed patients, we found that the culture-positive infection rates were not lower in the skin primarily-closed group as expected. Higher operative risk categories and early death rates of skin patch-closed ECMO patients (73% of skin patch-closed *vs.* 54% of the skin primarilyclosed patients died) might lead to underestimation of the diagnosis of infection in these patients. Besides prolonged ICU, mechanical ventilation and ECMO durations along with although not significant higher revision rates in skin primarilyclosed patients might cause infection rates higher than reported rates previously. Finally as not only BSI or mediastinitis but allcause infection rates of ECMO patients were demonstrated here, it would not be wise to explain infection of other sites only with the type of skin closure.

A longer duration of post-ECMO ventilation support in patients with culture-proven infection is another finding in our study in accordance with Bizarro et al.^[2]^ and Meyer et al.^[15]^ which was also reported to be a predictor for mortality for patients on ECMO support^[16]^.

Various reports have demonstrated different microorganisms cultured from patients on ECMO support^[17,18]^. *Coagulase-negative Staphylococci, Candida spp., Pseudomonas Aeruginosa* were three of the reported microorganisms responsible for the majority of positive cultures obtained from patients on ECMO support in the ELSO registry^[2]^, and *Candida spp*. was the most common cause in pediatric and adult age groups and the second most common agent in neonates.

*Candida spp., Coagulase-negative Staphylococci, Klebsiella, and Pseudomonas* were the most common isolates in our study. *Candida* species reported to be responsible for the majority of cases in pediatric ECMO patients were the most common isolated agent in our study in accordance with most studies^[2,4]^. The high prevalence of fungemia in ECMO patients might be due to the high severity of illness and longstanding use of wide-spectrum antibiotics before and during ECMO support^[19]^. Urinary tract infections due to *Candida* should be noted as an important part of nosocomial infections on ECMO support that has been demonstrated by other authors and highlighted in this study^[2,4,6,20]^. *Candida spp.* should be considered during an antimicrobial regimen selection when an infection is suspected in a patient on ECMO support.

A web-based survey^[21]^ of ELSO responses from 132 ELSO centers demonstrated that most centers administer antibiotic prophylaxis (mostly antibacterial) and almost half have a standardized protocol. Our institution does not have a standardized protocol for antibiotic prophylaxis, and our high infection prevalence might be partly due to being a new center with limited but increasing experience and a lack of standardized protocols.

In the pediatric population, ECMO has been widely used after cardiac surgery with an overall reported survival rate of 40-50% in different studies^[22-26]^. Although our ECMO survival was 68.2%, which is within the range reported by other studies^[27,28]^, the overall survival rate was 33.3%. The overall survival rate improved when the ECMO patients were evaluated in the means of years. The 24% overall survival in 2011-2012 increased to 39% in 20132014 following the switch to the DP3 system from the DP2 system after November 2012. Although improved, our overall survival rate was still lower than other reported rates^[29]^. No improvement in the culture positivity rates of ECMO patients was found between DP2 and DP3 systems. Several predictors of mortality were inconsistently identified from different studies, including cardiac arrest before ECMO, length of mechanical ventilation, infection/sepsis on ECMO, and duration of ECMO exceeding 8-10 days^[8,16,24,25,30]^. Besides in a study of 56 ECMO patients with congenital heart disease from Brazil, Miana et al.^[31]^ reported that after the ECMO program implementation with investment in training and equipment increased the probability of postcardiotomy ECMO weaning and survival. This low overall survival rate in our study might be due to induction of ECMO support for extended e-CPR or irreversible cardiac failure especially in the beginning period of ECMO support at our institution in 20112012 and partly due to factors such as infection on ECMO and increased length of mechanical ventilation.

### Limitation

This study has some limitations. Firstly, it was limited by its retrospective nature, data were collected by chart review and some laboratory data such as lactate or CRP levels were not available for the entire cohort. Secondly, our center is a tertiary cardiac center with a wide referral base for complex cardiac surgeries and the evaluation of patients were from a single center.

## CONCLUSION

In conclusion, ECMO is a life-saving modality in perioperative cardiac patients who have already increased risk for nosocomial infections. As a result, the only independent factor associated with nosocomial infection was prolonged ECMO support and although it was reported vice versa, the type of closure of skin did not have a substantial protective effect for infection in cardiac ECMO patients alone. The correction of the underlying process leading to ECMO support and shortening the length of ECMO duration together with stricter application of ECMO indications would be the best to prevent nosocomial infection in ECMO patients.

**Table:** 

Authors' roles & responsibilities	
PA	Conception and design of the work; acquisition of data; revising the work; approval of the final version
TK	Acquisition of data; drafting the paper; approval of the final version
EO	Analysis and interpretation of data; drafting the paper; revising the paper; approval of the final version
OY	Interpretation of data; revising the paper; approval of the final version
SO	Analysis and interpretation of data; drafting the paper; approval of the final version
YE	Interpretation of data; critical revising the work; approval of the final version
SH	Interpretation of data; revising the paper; approval of the final version
AG	Interpretation of data; revising the paper; approval of the final version

## References

[1] Bistrussu S, Beeton A, Castaldo G, Han J, Wong I, Tuleu C (2004). Are extracorporeal membrane oxygenation circuits that are primed
with plasmalyte and stored a likely source of
ınfection?. J Clin Microbiol.

[2] Bizzarro MJ, Conrad SA, Kaufman DA, Rycus P, Extracorporeal Life Support Organization Task Force on Infections,
Extracorporeal Membrane Oxygenation (2011). Infections acquired during extracorporeal membrane oxygenation in
neonates, children, and adults. Pediatr Crit Care Med.

[3] Brown KL, Ridout DA, Shaw M, Dodkins I, Smith LC, O'Callaghan MA (2006). Healthcare-associated infection in pediatric patients on
extracorporeal life support: the role of multidisciplinary
surveillance. Pediatr Crit Care Med.

[4] O'Neill JM, Schutze GE, Heulitt MJ, Simpson PM, Taylor BJ (2001). Nosocomial infections during extracorporeal membrane
oxygenation. Intensive Care Med.

[5] Horan TC, Andrus M, Dudeck MA (2008). CDC/NHSN surveillance definition of health care-associated
infection and criteria for specific types of infections in the acute care
setting. Am J Infect Control.

[6] Hsu MS, Chiu KM, Huang YT, Kao KL, Chu SH, Liao CH (2009). Risk factors for nosocomial infection during extracorporeal
membrane oxygenation. J Hosp Infect.

[7] Schutze GE, Heulitt MJ (1995). Infections during extracorporeal life support. J Pediatr Surg.

[8] Montgomery VL, Strotman JM, Ross MP (2000). Impact of multiple organ system dysfunction and nosocomial
infections on survival of children treated with extracorporeal membrane
oxygenation after heart surgery. Crit Care Med.

[9] (2010). Extracorporeal Life Support Organization Registry Report: International
Summary.

[10] Baslaim G, Bashore J, Al-Malki F, Jamjoom A (2006). Can the outcome of pediatric extracorporeal membrane oxygenation
after cardiac surgery be predicted?. Ann Thorac Cardiovasc Surg.

[11] Hocker JR, Wellhausen SR, Ward RA, Simpson PM, Cook LN (1991). Effect of extracorporeal membrane oxygenation on leukocyte
function in neonates. Artif Organs.

[12] Ide H, Kakiuchi T, Furuta N, Matsumoto H, Sudo K, Furuse A (1987). The effect of cardiopulmonary bypass on T cells and their
subpopulations. Ann Thorac Surg.

[13] Tajima K, Yamamoto F, Kawazoe K, Nakatani I, Sakai H, Abe T (1993). Cardiopulmonary bypass and cellular immunity: changes in
lymphocyte subsets and natural killer cell activity. Ann Thorac Surg.

[14] Nguyen DM, Mulder DS, Shennib H (1992). Effect of cardiopulmonary bypass on circulating lymphocyte
function. Ann Thorac Surg.

[15] Meyer DM, Jessen ME, Eberhart RC (1995). Neonatal extracorporeal membrane oxygenation complicated by
sepsis. Extracorporeal Life Support Organization. Ann Thorac Surg.

[16] Meliones JN, Custer JR, Snedecor S, Moler FW, O'Rourke PP, Delius RE (1991). Extracorporeal life support for cardiac assist in pediatric
patients. Review of ELSO Registry data. Circulation.

[17] Pieri M, Greco T, Scandroglio A, De Bonis M, Maj G, Fumagalli L (2012). Role of serum biomarkers in the diagnosis of infection in
patients undergoing extracorporeal membrane oxygenation. Crit Care.

[18] Hidron AI, Edwards JR, Patel J, Horan TC, Sievert DM, Pollock DA (2008). National Healthcare Safety Network Team; Participating National
Healthcare Safety Network Facilities. NHSN annual update:
antimicrobial-resistant pathogens associated with healthcare-associated
infections: annual summary of data reported to the National Healthcare
Safety Network at the Centers for Disease Control and Prevention,
2006-2007. Infect Control Hosp Epidemiol.

[19] Aubron C, Cheng AC, Pilcher D, Leong T, Magrin G, Cooper DJ (2013). Infections acquired by adults who receive extracorporeal membrane
oxygenation: risk factors and outcome. Infect Control Hosp Epidemiol.

[20] Coffin SE, Bell LM, Manning ML, Polin R (1997). Nosocomial ınfections in neonates receiving extracorporeal
membrane oxygenation. Infect Control Hosp Epidemiol.

[21] Kao LS, Fleming GM, Escamilla RJ, Lew DF, Lally KP (2011). Antimicrobial prophylaxis and infection surveillance in
extracorporeal membrane oxygenation patients: a multi-institutional survey
of practice patterns. ASAIO J.

[22] Chan T, Thiagarajan RR, Frank D, Bratton SL (2008). Survival after extracorporeal cardiopulmonary resuscitation in
infants and children with heart disease. J Thorac Cardiovasc Surg.

[23] Weinhaus L, Canter C, Noetzel M, McAlister W, Spray TL (1989). Extracorporeal membrane oxygenation for circulatory support after
repair of congenital heart defects. Ann Thorac Surg.

[24] Raithel SC, Pennington DG, Boegner E, Fiore A, Weber TR (1992). Extracorporeal membrane oxygenation in children after cardiac
surgery. Circulation.

[25] Black MD, Coles J G, Williams W G, Rebeyka IM, Trusler GA, Bohn D (1995). Determinants of success in pediatric cardiac patients undergoing
extracorporeal membrane oxygenation. Ann Thorac Surg.

[26] Conrad SA, Rycus PT, Dalton H (2005). Extracorporeal Life Support Registry Report 2004. ASAIO J.

[27] Flick RP, Sprung J, Gleich SJ, Barnes RD, Warner DO, Dearani JA (2008). Intraoperative extracorporeal membrane oxygenation and survival
of pediatric patients undergoing repair of congenital heart
disease. Paediatr Anaesth.

[28] Registry report of Extracorporeal Life Support Organization (1998). Ann Arbor: Extracorporeal Life Support Organization.

[29] Erek E, Haydin S, Onan B, Onan IS, Yazici P, Kocyigit O (2013). Extracorporeal life support experiences of a new congenital heart
center in Turkey. Artif Organs.

[30] Öztürk E, Yıldız O, Çine N, Tüzün B, Onan S, Ergül Y (2017). The use of neonatal extracorporeal life support in pediatric
cardiac intensive care unit. J Matern Fetal Neonatal Med.

[31] Miana LA, Caneo LF, Tanamati C, Penha JG, Guimarães VA, Miura N (2015). Post-cardiotomy ECMO in pediatric and congenital heart surgery:
impact of team training and equipment in the results. Rev Bras Cir Cardiovasc.

